# Nanoparticles of folic acid‐methyl‐β‐cyclodextrin (FA‐MβCD)/adamantane‐albumin exhibit enhanced antitumor activity compared with FA‐MβCD alone

**DOI:** 10.1002/2211-5463.13540

**Published:** 2022-12-26

**Authors:** Aiko Sakai, Yuki Yamashita, Shogo Misumi, Naoki Kishimoto, Risako Onodera, Taishi Higashi, Hidetoshi Arima, Keiichi Motoyama

**Affiliations:** ^1^ Graduate School of Pharmaceutical Sciences Kumamoto University Japan; ^2^ Priority Organization for Innovation and Excellence Kumamoto University Japan; ^3^ Laboratory of Evidence‐Based Pharmacotherapy Daiichi University of Pharmacy Fukuoka Japan

**Keywords:** anticancer drug, cyclodextrin, folate receptor, human serum albumin, supramolecular complex

## Abstract

Supramolecular drug carriers are a promising approach for delivering anticancer drugs with high blood retention after administration. We previously synthesized folic acid‐modified methyl‐β‐cyclodextrin (FA‐MβCD) as an anticancer drug. FA‐MβCD has a selective autophagy‐mediated antitumor effect on folic acid receptor (FR)‐expressing cancer cells. Here, we enhanced the antitumor effect and safety of FA‐MβCD by preparing a supramolecular nanoparticle formulation of FA‐MβCD via host–guest interactions using an adamantane conjugate with human serum albumin (Ad‐HSA). The Ad‐HSA/FA‐MβCD supramolecular complex prolonged the blood retention of FA‐MβCD and improved its antitumor effect and safety after intravenous administration in tumor‐bearing mice xenografted with FR‐expressing cancer cells. These results suggest that the supramolecular technique using Ad‐HSA is a promising approach for the delivery of CD‐based anticancer drugs.

## Graphical abstract

A supramolecular nanoparticle formulation of folic acid‐modified methyl‐β‐cyclodextrin (FA‐MβCD) by host–guest interactions using an adamantane conjugate with human serum albumin (Ad‐HSA). The Ad‐HSA/FA‐MβCD supramolecular complex significantly prolonged the blood retention of FA‐MβCD and improved its antitumor effect and safety after intravenous administration in tumor‐bearing nude mice xenografted with FR‐expressing cancer cells.

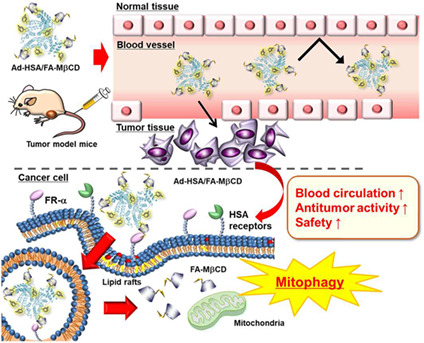

**Abbreviations**
Ad: adamantane
ALT: alanine aminotransferase
API: active pharmaceutical ingredient
AST: aspartate aminotransferase
BUN: blood urea nitrogen
CD: cyclodextrin
CRE: creatinine
DMT‐MM: 4‐(4,6‐dimethoxy‐1,3,5‐triazin‐2‐yl)‐4‐methylmorpholinium chloride
EPR: enhanced vascular permeability and retention
FA: folic acid
FR: folic acid receptor
HSA: human serum albumin
LC3: light chain 3
PEG: polyethylene glycol
ROS: reactive oxygen species
TRITC: tetramethylrhodamine isothiocyanate

Cyclodextrin (CD) is a cyclic oligosaccharide obtained by reacting starch with a CD‐producing enzyme, and it is classified as a host molecule that incorporates various drugs into its hydrophobic cavity to form an inclusion complex. The inclusion properties of CD have been exploited in various fields, such as food, cosmetics, clinical test agents, membrane science, and polymer chemistry. In the pharmaceutical field, the functionality of CD allows it to stabilize pharmaceutical products by the formation of complexes, enhancement of solubility, improvement of bioavailability, powderization of oily or low‐melting‐point substances, and prevention of volatilization, bitterness, and local irritation [1, 2, 3, 4, 5].

Recently, CD has been reported to function as a drug with certain pharmacological actions, and the paradigm of CD applications has shifted from excipients to active pharmaceutical ingredients (APIs). For example, several CD derivatives have been applied as APIs for the treatment of Niemann‐Pick disease type C [6, 7, 8], familial amyloid polyneuropathy [9, 10], cancer [11, 12, 13, 14, 15], and septic shock [16] and as an adjuvant [17, 18, 19]. Grosse et al. [20, 21] previously reported that intraperitoneal administration of methyl‐β‐CD (MβCD) in cancer‐bearing mice has a higher antitumor activity than doxorubicin alone. However, MβCD does not have the ability to target tumor cells. Therefore, to improve the tumor selectivity of MβCD, we modified MβCD with folic acid (FA), a tumor‐targeting ligand and evaluated its folate receptor (FR)‐mediated anticancer activity. FA‐modified MβCD (FA‐MβCD) was selectively taken up by FR‐expressing cancer cells and provided great cytotoxic activity via autophagy induction [13, 14]. In addition, FA‐MβCD suppressed ATP production and enhanced reactive oxygen species (ROS) production in FR‐expressing KB cells [11]. Importantly, FA‐MβCD enhanced the conversion of light chain 3‐I (LC3‐I) to LC3‐II in the FR‐expressing KB cells. In addition, FA‐MβCD elevates the expression of PINK1, a marker protein of mitophagy [11]. FA‐MβCD significantly suppressed tumor growth in mice inoculated with FR‐expressing KB cells at a dose of 20 mg·kg^−1^ [11]. However, our preliminary studies revealed that the half‐life of FA‐MβCD was approximately 2.5 min, suggesting that it disappeared from the blood immediately after intravenous administration. FA‐MβCD was presumed to have a molecular weight of ca. 1738 Da, a particle size of several nanometers, and a water‐soluble property; therefore, it was rapidly excreted by the kidney. In addition, FA‐MβCD did not show any significant antitumor effects at a single dose of 1 mg·kg^−1^ or less. Therefore, repeated administration is required for FA‐MβCD to exert sufficient antitumor activity at low doses. Based on this background, we hypothesized that increasing the molecular weight of FA‐MβCD can improve its retention in the blood and accumulation in tumors, thereby resulting in enhanced antitumor effects and reduced side effects.

To obtain a drug with high molecular weight, the attachment of polymers or proteins to small‐molecule drugs is a promising approach. For example, polyethylene glycol (PEG) [22], copolymers [23], transferrin [24, 25], and human serum albumin (HSA) [26, 27] are often used to increase the molecular weights of drugs. These macromolecules are attached to small‐molecule drugs primarily by covalent bonds; however, the loss of drug activity due to changes in conformation is a serious problem. Thus far, we have developed a “Self‐assembly PEGylation Retaining Activity (SPRA)” technique to modify PEG to insulin through host–guest interactions between CD and adamantane (Ad), and this insulin supramolecular complex prepared using SPRA technology succeeded in increasing blood retention without loss of insulin activity [28]. Therefore, it would be possible to bind macromolecules to FA‐MβCD via supramolecular complexation via the host–guest interaction of SPRA technology.

Human serum albumin has attracted attention as a drug carrier in clinical practice, and HSA‐based drug delivery formulations have been developed [29]. Several anticancer drug formulations are used in combination with HSA, such as methotrexate‐HSA conjugate [30], HSA‐bound prodrug using doxorubicin [29], and albumin paclitaxel nanoparticles (Abraxane^®^) [31, 32]. In particular, Abraxane^®^ has been approved for the treatment of breast cancer, gastric cancer, non‐small cell lung cancer, and unresectable pancreatic cancer. Therefore, we selected HSA as the carrier macromolecule for FA‐MβCD.

In this study, we first attached Ad to HSA (Ad‐HSA) as a drug carrier with excellent safety and blood retention. Next, we prepared an Ad‐HSA/FA‐MβCD supramolecular complex and examined whether it could improve blood retention, antitumor effects, and safety.

## Materials and methods

### Materials

Adamantane acetate was purchased from the Tokyo Chemical Industry (Tokyo, Japan). Human serum albumin (HSA) was purchased from Nacalai Tesque (Kyoto, Japan). Tetramethylrhodamine isothiocyanate (TRITC) was purchased from Funakoshi (Tokyo, Japan). The Cyto‐ID^®^ Autophagy Detection Kit and Mitophagy Detection Kit were purchased from Enzo Life Sciences (Farmingdale, NY, USA) and Dojindo (Kumamoto, Japan), respectively. MitoTracker^®^ Green and LysoTracker^®^ Green were obtained from Invitrogen (Carlsbad, CA, USA).

### Synthesis of adamantane‐modified human serum albumin (Ad‐HSA)

1‐Adamantane acetic acid (29.2 mg), HSA (100 mg), and the condensing agent 4‐(4,6‐dimethoxy‐1,3,5‐triazin‐2‐yl)‐4‐methylmorpholinium chloride (DMT‐MM, 56 mg in 500 μL of MeOH) were dissolved in water (10 mL) and stirred at room temperature for 24 h. Unreacted 1‐adamantane acetic acid was removed by dialysis (Spectra/Por^®^ Membrane MWCO: 8000, solvent : water). The purified Ad‐HSA was obtained by freeze‐drying.

### Cells

KB cells, a subline of HeLa cells, were obtained from the Institute of Development, Aging and Cancer, Tohoku University (Sendai, Japan). A549 human lung carcinoma cells were purchased from American Type Culture Collection (Rockville, MD, USA).

### *In vitro* antitumor activity

*In vitro* antitumor activity was determined using the WST‐1 method. Briefly, KB cells or A549 cells were seeded on a 96‐well culture plate at 2 × 10^4^ cells/well and cultured in a medium containing 10% FBS for 24 h. After washing the cells twice with 150 μL of medium, FA‐MβCD (5 mm) or the Ad‐HSA (1.25 mm)/FA‐MβCD (5 mm) complex was added and incubated for 2 h. Then, cell viability was assayed by the WST‐1 method as reported previously [11]. Cell viability was calculated assuming that the absorbance of the serum‐free medium containing Tween 20 (1% (v/v)) and the sample‐free system were 0% and 100%, respectively.

### Cellular localization

KB cells were seeded on a 35 mm glass bottom dish at 2 × 10^5^ cells/dish and cultured in a medium containing 10% FBS for 24 h. After washing the cells with PBS, 150 μL of serum‐free medium containing TRITC‐FA‐MβCD (10 μm) or Ad‐HSA (2.5 μm)/TRITC‐FA‐MβCD (10 μm) was added, and the cells were cultured at 37 °C for 2 h. LysoTracker^®^ (final concentration 100 nm), an acidic organelle marker, was then added. After further incubation for 30 min, the cells were washed with 1 mL of serum‐free medium, and the fluorescence of TRITC and LysoTracker^®^ was observed using a confocal laser scanning microscope.

### Autophagosome formation

KB cells (3.5 × 10^5^ cells/dish) on a 35 mm glass bottom dish were treated with 150 μL of FA‐MβCD (5 mm) or Ad‐HSA (1.25 mm)/FA‐MβCD (5 mm) and incubated at 37 °C for 2 h. Then, after washing twice with serum‐free medium, 1 mL of Cyto‐ID^®^ solution was added. After incubation at 37 °C for 30 min, fluorescence images were obtained using a fluorescence microscope (Biozero BZ‐8000; Keyence, Osaka, Japan).

### Detection of mitophagy

Mitophagy was detected in KB cells using a Mitophagy Detection Kit^®^ (Dojindo, Kumamoto, Japan) as reported previously [11]. KB cells (3.5 × 10^5^/35 mm glass bottom dish) were treated with 5 mm MβCD for 2 h. Mitophagy in the cells was visualized by the Mitophagy Detection Kit^®^ and scanned with a Biozero BZ‐8000 fluorescence microscope (Keyence).

### Antitumor activity *in vivo*

KB cell suspension (1 × 10^6^ cells/100 μL) was subcutaneously inoculated into BALB/c nu/nu mice (male, 4‐weeks‐old, and ca. 20 g). Fourteen days later, 1 mg·kg^−1^ FA‐MβCD or the Ad‐HSA (9.7 mg·kg^−1^)/FA‐MβCD (1 mg·kg^−1^) complex was intravenously administered to the mice once a week. The tumor volumes were determined as reported previously [33]. Thirty days after the first intravenous administration of the samples, the blood chemistry data of aspartate aminotransferase (AST), alanine aminotransferase (ALT), creatinine (CRE), and blood urea nitrogen (BUN) in the serum were analyzed using a JCA‐BM2250 clinical chemistry analyzer (JEOL, Tokyo, Japan). The *in vivo* experiments were performed with the approval of the Ethics Committee of Kumamoto University (approval ID:24‐286).

### Plasma level of FA‐MβCD

The Ad‐HSA/FA‐MβCD complex was administered via single intravenous administration to KB cell‐bearing mice. Blood was collected at 5, 10, 30, 60, and 180 min after intravenous administration. After centrifugation at 1000 ***g*** for 15 min, the supernatant was collected and the proteins were removed using Amicon Ultra (MERCK, Darmstadt, Germany; UFC UFC501024, MWCO: 10 kDa). The FA‐MβCD levels in the plasma samples were determined by HPLC. The HPLC conditions were as follows: column: COSMOSIL 5C18‐MS‐II (4.6 mm i.d. × 150 mm, 5 μm); mobile phase: MeOH/H_2_O = 1/9; detection wavelength: Ex 360 nm and Em 450 nm; injection volume: 20 μL; and flow rate: 1.0 mL·min^−1^.

### Statistics

Scheffe's test was performed using statview software (Abacus Corporation, Berkeley, CA, USA), and a value of *P* < 0.05 was considered significant. The number of experiments is shown in the figure legends. Graph data are presented as the mean ± SEM.

## Results and Discussion

### Fabrication of the Ad‐HSA conjugate

**Fig. 1:**
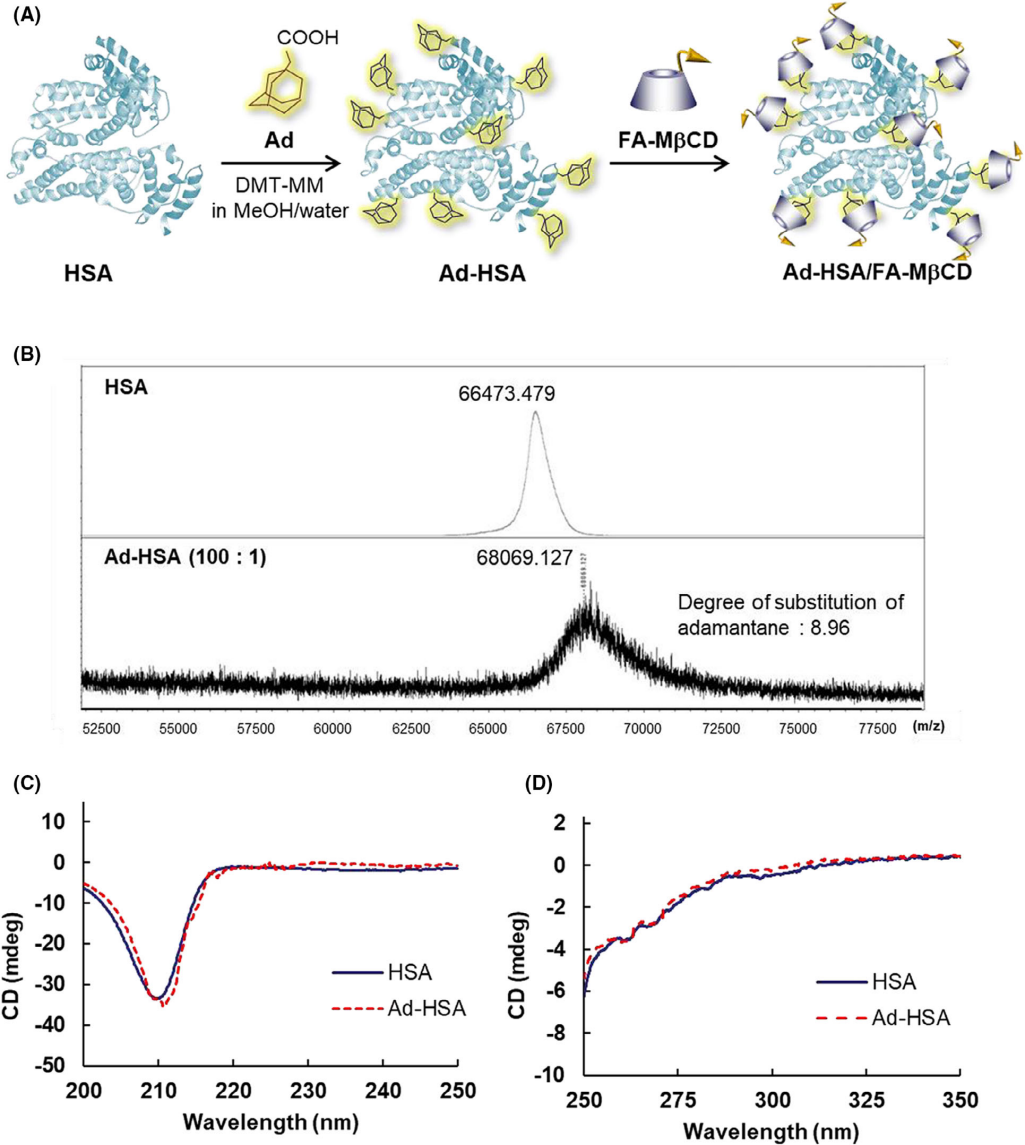
Preparation pathway of Ad‐HSA (A), MALDI‐TOF MS spectrum (B), and circular dichroism spectra of Ad‐HSA (C, D). Concentration of HSA and Ad‐HSA was 15 μm in PBS (pH 7.4). Circular dichroism spectra of Ad‐HSA are shown in the far‐ultraviolet field (C) and near‐ultraviolet field (D).

Figure 1A shows the synthesis pathway for the Ad‐HSA conjugate. After dissolving HSA and 1‐adamantane acetic acid in methanol/water, the condensing agent DMT‐MM was added, and the mixture was stirred for 24 h. Finally, the resulting Ad‐HSA conjugate was purified by dialysis. Figure 1B shows the MALDI‐TOF‐MS spectra of HSA alone and the Ad‐HSA conjugate. HSA exhibited a major peak at a mass number of 66 473.5 (*m/z*). In contrast, the Ad‐HSA conjugate prepared at a molar ratio of 1 : 100 exhibited a major peak at a mass number of 68 069.1 (*m/z*), suggesting the formation of a conjugate with an average degree of substitution of Ad of 9.

When chemically modifying a protein, it is important to investigate its structure because inactivation of the parent protein and immune response may occur due to structural changes. Although Ad is a small molecule (MW: 194 Da), it is a hydrophobic molecule that may affect the three‐dimensional structure of HSA. Therefore, the three‐dimensional structure of the synthesized Ad‐HSA conjugate was examined using circular dichroism spectra. Figure 1C,D show the circular dichroism spectra of the HSA and Ad‐HSA conjugates in the far‐ultraviolet and near‐ultraviolet regions, respectively. In both regions, the circular dichroism spectrum of the Ad‐HSA conjugate was similar to that of HSA. These results show that the modification of Ad did not change the three‐dimensional structure of HSA.

### Physicochemical properties of Ad‐HAS/FA‐MβCD complex

Although enhanced vascular permeability and retention (EPR) effects are still controversial concepts, certain molecules, such as macromolecular drugs, nanoparticles, and liposomes, tend to accumulate in solid tumor tissues. In general, for a nanoparticle formulation to obtain EPR effects, the particle size of the formulation must be 200 nm or less and it must be charge‐neutral [34]. Therefore, the particle size and ζ‐potential of the Ad‐HSA conjugate and Ad‐HSA/FA‐MβCD complex were measured. As shown in Table 1, the particle size of HSA alone was 14.5 nm, whereas that of Ad‐HSA alone was 161 nm. In contrast, the particle size of the Ad‐HSA/FA‐MβCD complex decreased to ca. 79.6 nm. In addition, the ζ‐potential of the Ad‐HSA/FA‐MβCD complex was near‐neutral (−0.12 mV). Leamon et al. [35] reported that when the ζ‐potential of the FA‐modified carrier was closer to neutral, non‐specific adsorption on the cell surface was reduced and recognition of FR was increased. Furthermore, Reddy and Low [36] reported that the particle size of FA‐modified carriers taken up by FR‐mediated endocytosis was ≤ 150 nm or less. Taken together, these results suggest that the particle size and ζ‐potential of the Ad‐HSA/FA‐MβCD complex are suitable for the EPR effects and endocytosis via FR.

**Table 1:** Particle sizes and ζ‐potentials of Ad‐HSA and Ad‐HSA/FA‐MβCD.

System	Mean diameter (nm)	ζ‐Potential (mV)
HSA alone	14.5 ± 0.59	–
Ad‐HSA alone	161 ± 11.2	−2.76 ± 1.21
Ad‐HSA/FA‐MβCD	79.6 ± 6.09	−0.12 ± 0.26

Particle sizes and ζ‐potentials were determined using a Zetasizer Nano. The samples were dissolved in a 5% mannitol solution (pH 7.4). The concentrations of HSA and Ad‐HSA were 0.5 mg·mL^−1^. The molar ratio of Ad‐HSA/FA‐MβCD was 1/4.

### Cytotoxic activity of Ad‐HSA/FA‐MβCD complex

**Fig. 2:**
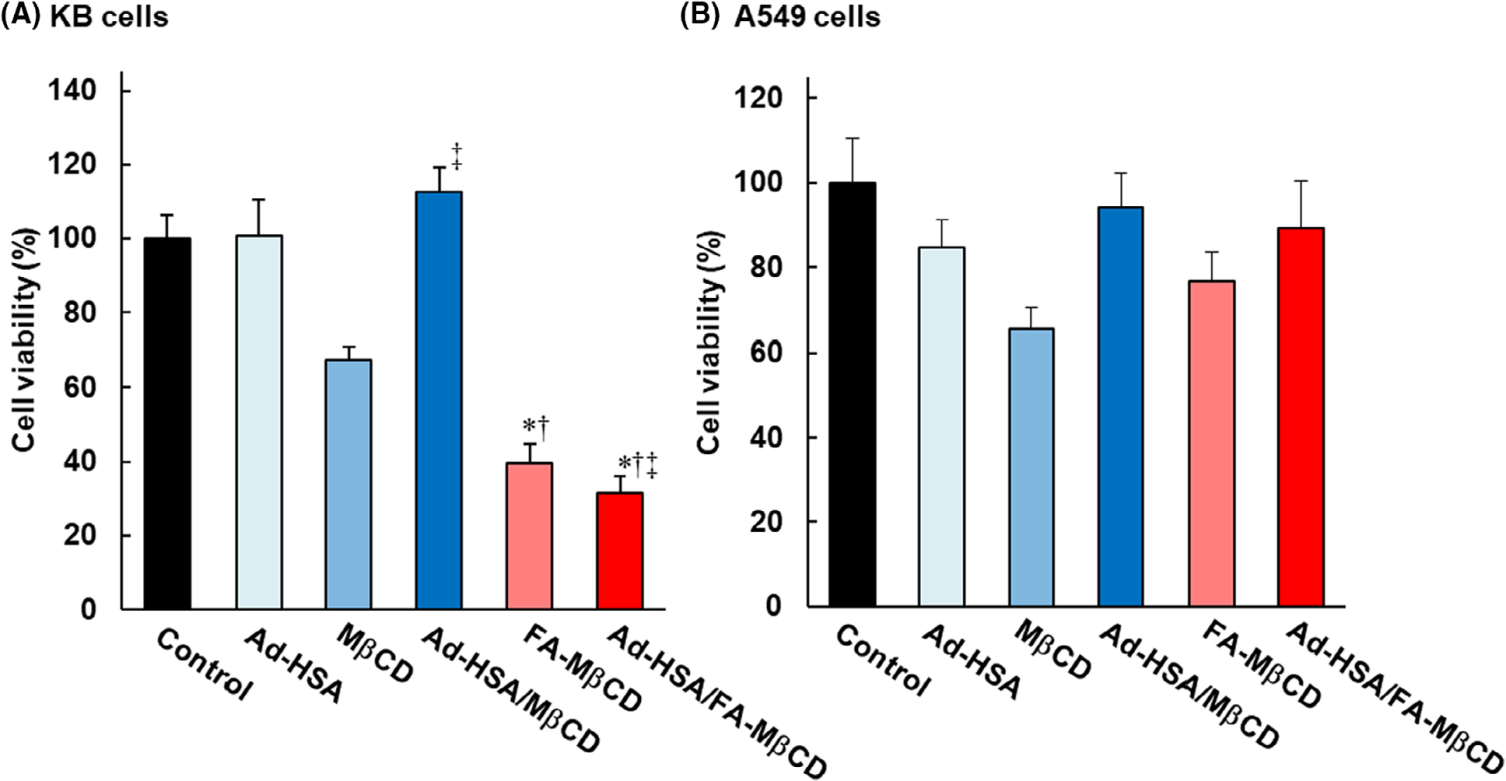
Cytotoxic activity of Ad‐HSA/FA‐MβCD for (A) KB cells and (B) A549 cells. KB or A549 cells were incubated with Ad‐HSA/FA‐MβCD (5 mm) at 37 °C for 2 h. Data are analyzed by Scheffe's test and the Mean ± SEM are shown (*n* = 3); *P < 0.05, compared with the control, ^†^ *P* < 0.05, compared with Ad‐HAS, and ^‡^ *P* < 0.05, compared with MβCD.

For the Ad‐HSA/FA‐MβCD complex to exhibit a cytotoxic effect on tumor cells, FA‐MβCD was dissociated from the Ad‐HSA conjugate. Therefore, we investigated whether the Ad‐HSA/FA‐MβCD complex exhibited cytotoxic effects using the WST‐1 method. KB cells (FR‐expressing cells, FR (+)) and A549 cells (FR non‐expressing cells, FR (−)) were used to examine cytotoxicity. Figure 2A shows the viability of KB cells (FR (+)) after treatment with the Ad‐HSA/FA‐MβCD complex. The Ad‐HSA/MβCD complex attenuated the cytotoxic effects of MβCD. The Ad‐HSA/FA‐MβCD complex showed cytotoxic effects similar to those of FA‐MβCD alone. In contrast, FA‐MβCD alone and the Ad‐HSA/FA‐MβCD complex showed no significant cytotoxic effects on A549 cells (FR (−)) (Fig. 2B). These results suggested that the Ad‐HSA/FA‐MβCD complex exhibited cytotoxic effects similar to those of FA‐MβCD alone in FR‐expressing cancer cells.

### Cellular uptake of Ad‐HSA/FA‐MβCD complex

**Fig. 3:**
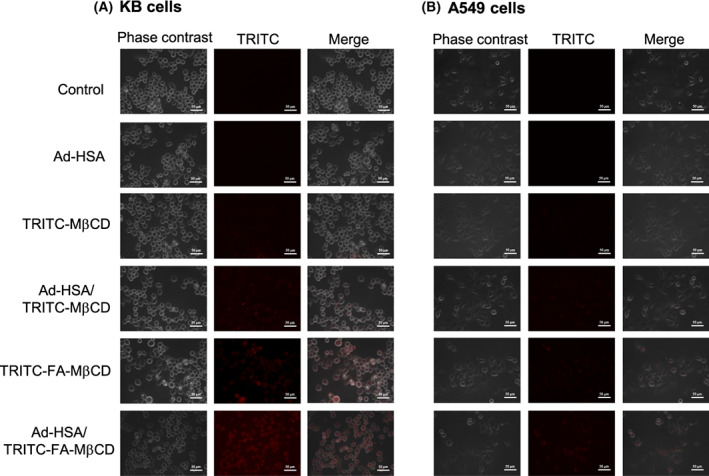
Cellular uptake of the Ad‐HSA/TRITC‐FA‐MβCD complex in (A) KB cells and (B) A549 cells. KB or A549 cells were treated with Ad‐HSA/TRITC‐FA‐MβCD (2.5 μm/10 μm) for 2 h. Representative images are presented (*n* = 3). Scale bar: 50 μm.

Next, we investigated the intracellular dynamics of the Ad‐HSA/FA‐MβCD complexes. KB cells (FR (+)) or A549 cells (FR (−)) were treated with a complex of Ad‐HSA and TRITC‐labeled FA‐MβCD, and the fluorescence derived from TRITC was observed using a fluorescence microscope (Fig. 3). The Ad‐HSA/TRITC‐FA‐MβCD complex was taken up by KB cells (FR (+)), and its uptake may be higher than that of TRITC‐FA‐MβCD alone (Fig. 3A). TRITC‐MβCD alone was not taken up by KB cells (FR (+)), whereas uptake of the Ad‐HSA/TRITC‐MβCD complex was observed. In A549 cells (FR (−)), TRITC‐MβCD alone and TRITC‐FA‐MβCD alone were not taken up by the cells, whereas their complexes were slightly taken up by the cells (Fig. 3B).

The HSA receptors FcRn (neonatal Fc receptor) and SPARC (secreted protein acidic and rich in cysteine) are expressed in KB cells and A549 cells [37, 38]. Since the conformation of the Ad‐HSA conjugate is similar to that of HSA (Fig. 1C,D), the Ad‐HSA/FA‐MβCD complex may also be recognized by HSA receptors and incorporated into the cells. Collectively, these results suggest that the Ad‐HSA/FA‐MβCD complex is taken up by the cell via FR or HSA receptors.

**Fig. 4:**
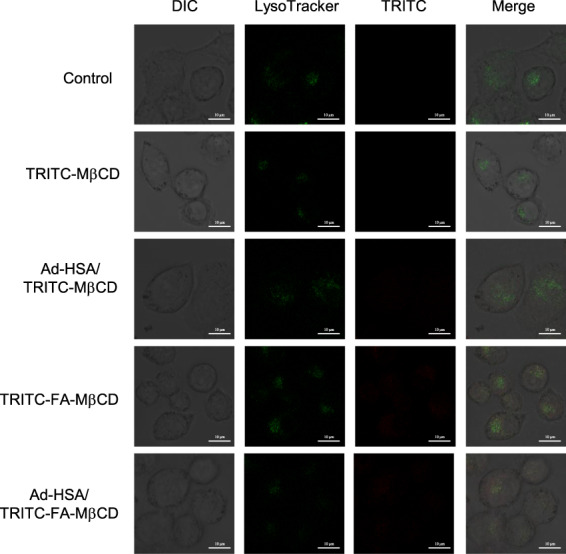
Cellular localization of Ad‐HSA/TRITC‐FA‐MβCD in KB cells. KB cells were treated with Ad‐HSA/TRITC‐FA‐MβCD (2.5 μm/10 μm) for 2 h, and then treated with LysoTracker®. Representative images are presented (*n* = 3). Scale bar: 10 μm.

For FA‐MβCD to exhibit antitumor effects, it must be taken up into tumor cells, escape endosomes, and translocate to the mitochondria. Therefore, we investigated the endosomal escape ability of the Ad‐HSA/FA‐MβCD complexes. KB cells (FR (+)) were treated with the Ad‐HSA/TRITC‐FA‐MβCD complex, and the intracellular endolysosomes were stained with LysoTracker^®^. In the Ad‐HSA/TRITC‐FA‐MβCD complex system, part of TRITC‐FA‐MβCD co‐localized with endolysosomes, although the majority was widely distributed throughout the cells (Fig. 4). In addition, no significant difference was observed in the intracellular distribution of TRITC fluorescence between TRITC‐FA‐MβCD alone and the Ad‐HSA/TRITC‐FA‐MβCD complex. In contrast, in the Ad‐HSA/TRITC‐MβCD complex, most TRITC‐MβCD tended to co‐localize with endolysosomes. These results suggest that the Ad‐HSA/FA‐MβCD complex can escape endosomes after uptake into cells.

### Autophagy induction by the Ad‐HSA/FA‐MβCD complex

**Fig. 5:**
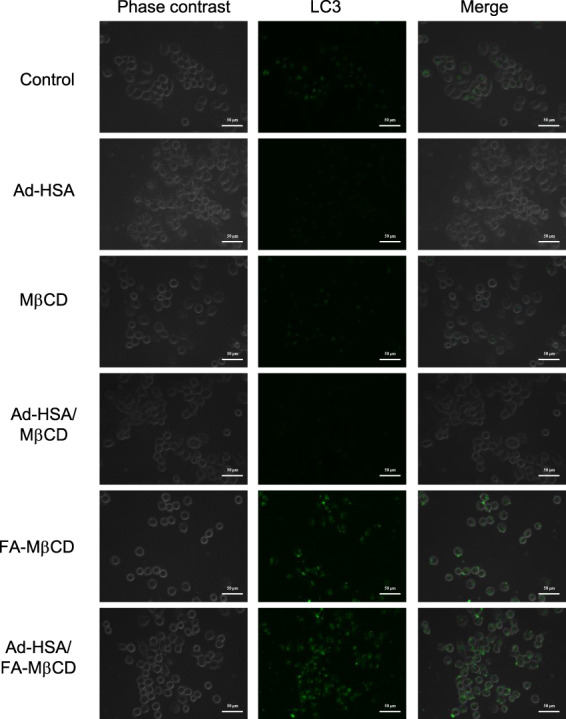
Autophagosome formation in KB cells by the Ad‐HSA/FA‐MβCD complex. KB cells were treated with Ad‐HSA/FA‐MβCD (5 mm) for 2 h, followed by treatment with Cyto‐ID® for 30 min. Representative images are presented (*n* = 3). Scale bar: 50 μm.

Autophagy is known to have the dual effect of promoting and suppressing cancer [39]. In cells with autophagy deficiency, mitochondrial dysfunction and genomic instability occur and tumorigenesis is promoted. In other words, tumorigenesis was suppressed by increased autophagy. Moreover, since the tumor tissues that have grown and progressed to some extent are in a state of low nutrition and low blood flow, the tumor cells activate autophagy and supplement the nutritional deficiency; therefore, the regulation of autophagy in cancer is important. The therapeutic efficacy of drugs for promoting or inhibiting autophagy has been investigated in various cells, and clinical trials have been conducted [40]. To date, we have reported that FA‐MβCD induces autophagy‐mediated cell death in KB cells (FR (+)) [11, 15]. Therefore, we investigated whether the Ad‐HSA/FA‐MβCD complex induced autophagy in KB cells (FR (+)) using the Cyto‐ID^®^ autophagy detection kit. As shown in Fig. 5, the Ad‐HSA/FA‐MβCD complex showed increased autophagosome‐derived fluorescence in KB cells (FR (+)) compared with the control group. In contrast, autophagosome formation was not observed in the Ad‐HSA/MβCD complex‐treated cells. These results suggest that the Ad‐HSA/FA‐MβCD complex induces autophagy in the same manner as FA‐MβCD.

**Fig. 6:**
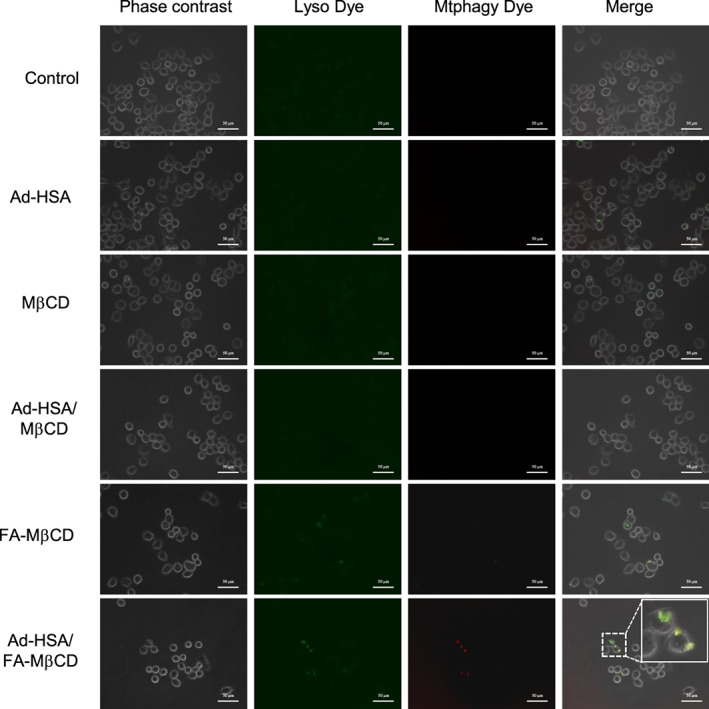
Mitophagy induction in KB cells by the Ad‐HSA/MβCD complex. KB cells were treated Mtphagy Dye® for 15 min at 37 °C. The cells were then treated with Ad‐HSA/MβCD (1.25 mm/5 mm) for 2 h and then Lyso Dye® for 10 min at 37 °C. Representative images are presented (*n* = 3). Scale bar: 50 μm.

Mitophagy contributes to cellular survival via the removal of damaged mitochondria, thereby eliminating the source of apoptogenic signals and excessive ROS. Therefore, regulation of mitophagy in tumor tissues is a promising approach in cancer treatment. We previously reported that FA‐MβCD induces mitophagy in KB cells (FR (+)) [11]. Therefore, we investigated whether the Ad‐HSA/FA‐MβCD complex could induce mitophagy in cells using Mtphagy Dye^®^, a mitophagy detection reagent. Mtphagy Dye^®^ permeates the living cell membrane, accumulates in intracellular mitochondria, and is then immobilized on the mitochondria by chemical bonds. Normally, the fluorescence intensity of Mtphagy Dye^®^ is low; however, when mitophagy is induced and mitochondria fuse with lysosomes, the fluorescence intensity of Mtphagy Dye^®^ increases significantly. KB cells (FR (+)) were pretreated with Mtphagy Dye^®^ and then treated with the Ad‐HSA/FA‐MβCD complex. The lysosomes were then stained with Lyso Dye^®^ and observed under a fluorescence microscope. As shown in Fig. 6, red fluorescence from Mtphagy Dye^®^ was observed in the cells treated with FA‐MβCD and the Ad‐HSA/FA‐MβCD complexes. In contrast, fluorescence from Mtphagy Dye^®^ was not observed in KB cells (FR (+)) treated with the Ad‐HSA/MβCD complex. In addition, to determine mitochondrial function after treatment with the Ad‐HSA/MβCD complex, we examined ATP and mitochondrial ROS (mtROS) production in KB cells (FR (+)). The Ad‐HSA/MβCD complex suppressed ATP production and enhanced mtROS production, which was similar to FA‐MβCD, suggesting that the complex impaired mitochondrial function in KB cells (FR (+)) (data not shown). Taken together, these results suggested that the Ad‐HSA/FA‐MβCD complex induced mitophagy.

### Antitumor activity of the Ad‐HSA/FA‐MβCD complex

**Fig. 7:**
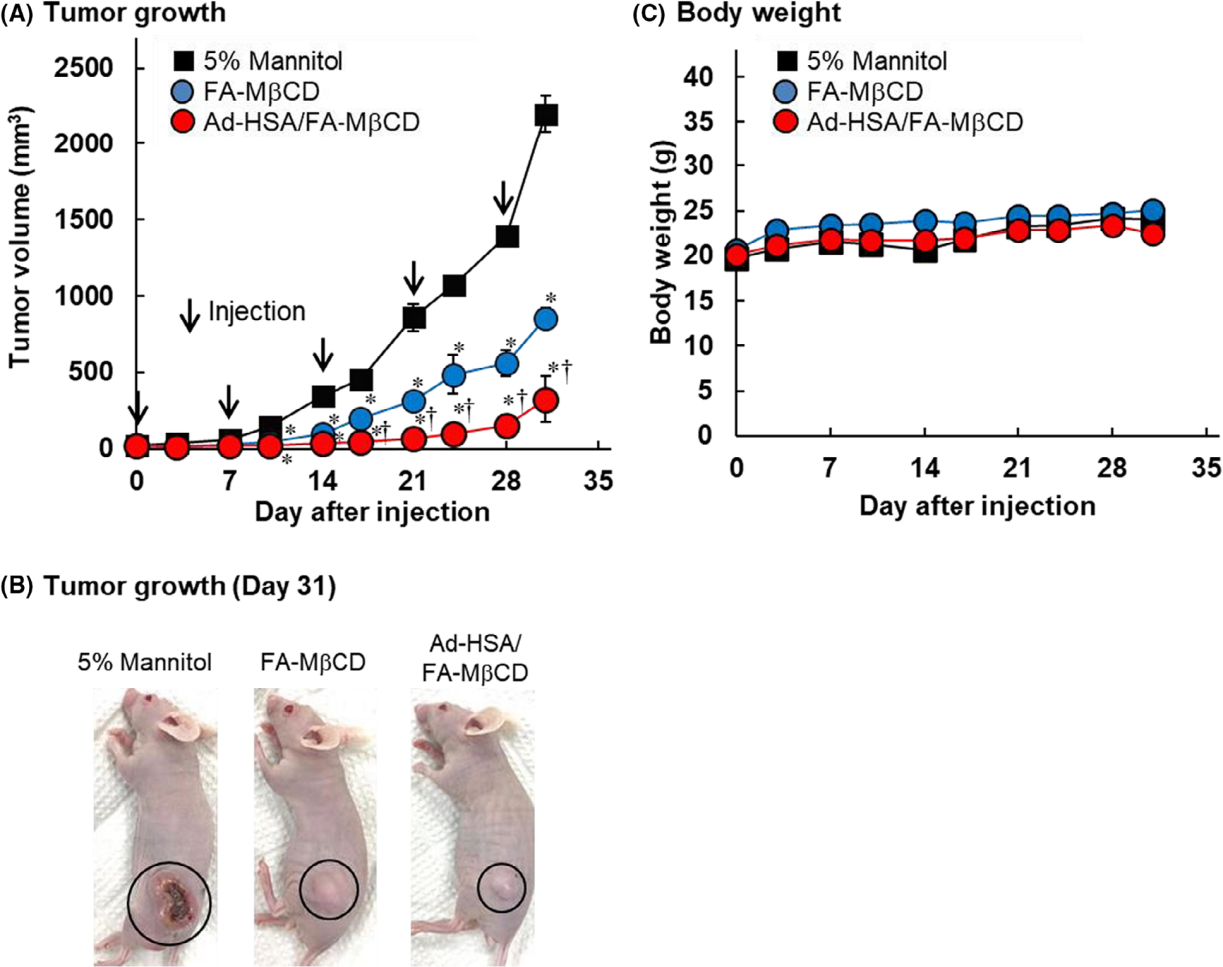
Effects of the Ad‐HSA/FA‐MβCD complex on tumor growth (A, B) and body weight (C) in mice bearing KB cells. FA‐MβCD (1 mg·kg^−1^) or the Ad‐HSA (9.7 mg·kg^−1^)/FA‐MβCD (1 mg·kg^−1^) complex was administered by a single intravenous injection to KB cell‐bearing mice. Data are analyzed by Scheffe's test and the means ± SEM are shown (*n* = 4–5); *P < 0.05, compared with the control (5% mannitol), and ^†^ *P* < 0.05, compared with FA‐MβCD.

We investigated the antitumor activity of the Ad‐HSA/FA‐MβCD complex after intravenous administration to tumor‐bearing nude mice xenografted with KB cells (FR (+)). Figure 7 shows the changes in tumor volume and body weight after repeated administration of the Ad‐HSA (9.7 mg·kg^−1^)/FA‐MβCD (1 mg·kg^−1^) complex once a week for up to 28 days. The Ad‐HSA/FA‐MβCD complex significantly suppressed tumor growth compared with the control group (5% mannitol) and FA‐MβCD alone (Fig. 7A,B). In addition, no difference in body weight was observed among the groups (Fig. 7C). These results suggest that the Ad‐HSA/FA‐MβCD complex significantly enhanced the antitumor effect of FA‐MβCD.

To evaluate the safety of the Ad‐HSA/FA‐MβCD complex, we analyzed blood chemistry data at the endpoint of the antitumor study. As shown in Table 2, repeated administration of FA‐MβCD alone increased the serum AST and ALT levels, suggesting that long‐term repeated administration may induce liver damage. The liver is the central organ responsible for the metabolism of the living body, and various organelles in hepatocytes, such as mitochondria, microsomes, and peroxisomes, undergo enzymatic reactions, resulting in high ROS production. Mitochondria play the most important role as an energy source, and they simultaneously consume a high amount of oxygen and produce a large amount of ROS. Oxidative stress in the liver suppresses hepsidin production, which suppresses iron absorption. The large amount of iron accumulation generates excessive hydroxyl radicals via the Fenton reaction and induces liver damage [41]. In contrast, we have previously reported that FA‐MβCD enhances ROS production through mitochondrial dysfunction [11]. Therefore, a portion of FA‐MβCD is transferred to the liver by repeated administration and induces chronic ROS accumulation in hepatocytes and liver damage. In contrast, repeated administration of the Ad‐HSA/FA‐MβCD complex did not increase the serum AST or ALT levels (Table 2). These results suggest that the Ad‐HSA/FA‐MβCD complex is safer than FA‐MβCD.

**Table 2:** Blood chemistry data after repeated administration of Ad‐HSA/FA‐MβCD in mice bearing KB cells.

System	CRE (mg·dL^−1^)	BUN (mg·dL^−1^)	AST (mg·dL^−1^)	ALT (mg·dL^−1^)
5% Mannitol	0.30 ± 0.04	21.3 ± 1.38	52.0 ± 1.87	15.8 ± 0.63
FA‐MβCD alone	0.20 ± 0.09	17.4 ± 2.64	91.3 ± 27.6*	166.7 ± 72.0*
Ad‐HSA/FA‐MβCD	0.13 ± 0.03	18.2 ± 2.74	52.0 ± 2.42^†^	22.8 ± 2.29^†^

* *P* < 0.05, compared with the control (5% mannitol solution)

^†^ *P* < 0.05, compared with FA‐MβCD.

* *P* < 0.05, compared with the control (5% mannitol solution)

Serum was collected 30 days after the first intravenous administration of FA‐MβCD (1 mg·kg^−1^) or Ad‐HSA (9.7 mg·kg^−1^)/FA‐MβCD (1 mg·kg^−1^) complex. Means ± SEM are shown (*n* = 3–4).

**Fig. 8:**
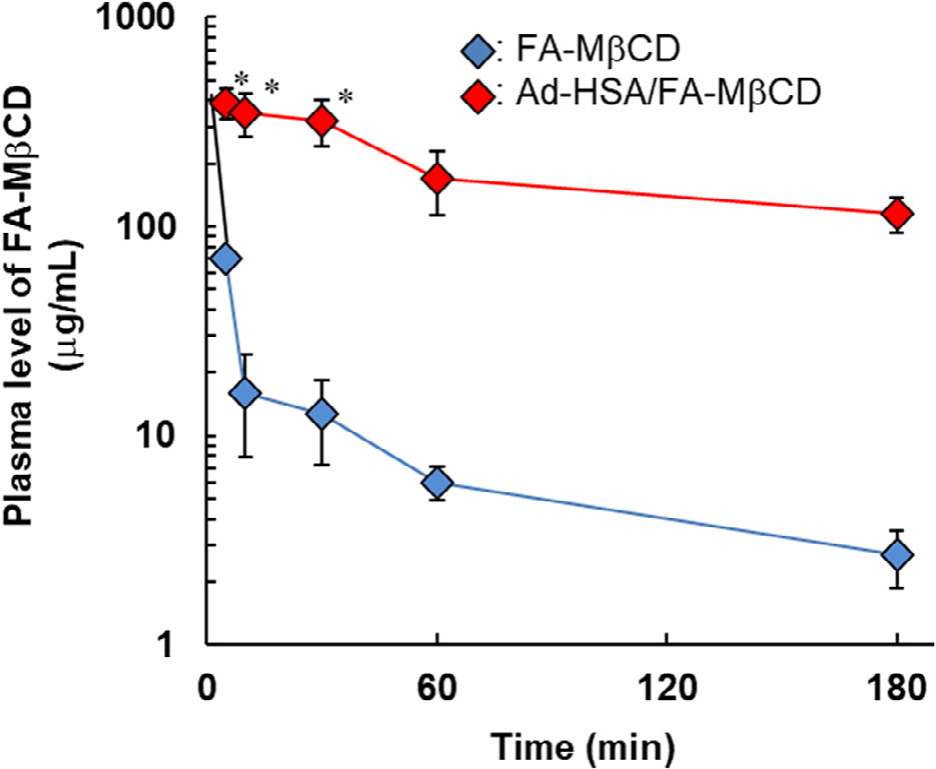
Plasma level of FA‐MβCD after intravenous administration of Ad‐HSA/FA‐MβCD complex in mice bearing KB cells. FA‐MβCD (1 mg·kg^−1^) or Ad‐HSA (9.7 mg·kg^−1^)/FA‐MβCD (1 mg·kg^−1^) complex was administered by a single intravenous route in KB cell‐bearing mice. Data are analyzed by Scheffe's test and the Means ± SEM are shown (*n* = 3–4); *P < 0.05, compared with FA‐MβCD.

Next, we examined the plasma levels of FA‐MβCD after a single intravenous administration of the complex to tumor‐bearing mice (Fig. 8). In the Ad‐HSA/FA‐MβCD complex system, approximately 30% of the FA‐MβCD dose remained in the blood at 3 h after intravenous administration. In addition, the half‐life of FA‐MβCD in the complex system was approximately 80.2 min, which was approximately 32 times higher than that of FA‐MβCD alone (Table 3). The elimination constants calculated from the plasma levels in the Ad‐HSA/FA‐MβCD complex and FA‐MβCD alone were approximately 0.010 and 0.327 min^−1^, respectively (Table 3). These results suggest that the Ad‐HSA/FA‐MβCD complex can improve blood retention of FA‐MβCD.

**Table 3:** Pharmacokinetic parameters.

System	*k*_e_ (min^−1^)	*t*_1/2_ (min)
FA‐MβCD alone	0.327 ± 0.040	2.5 ± 0.38
Ad‐HSA/FA‐MβCD	0.010 ± 0.003	80.2 ± 23.1

*t*_1/2_, half‐life; *k*_e_, elimination constant.

The contribution of the EPR effect likely explains why the Ad‐HSA/FA‐MβCD complex enhances the antitumor effect of FA‐MβCD after intravenous administration. In fact, the particle size of the Ad‐HSA/FA‐MβCD complex was approximately 79.6 nm and the ζ‐potential was −0.12 mV (Table 1); therefore, this complex has suitable nanoparticle properties for the EPR effect. In addition, antitumor effects were not observed with FA‐unmodified MβCD alone, whereas slight antitumor effects were observed with the Ad‐HSA/FA‐unmodified MβCD complex (data not shown). Therefore, a supramolecular complex system using Ad‐HSA is a promising approach for enhancing CD‐based antitumor drugs.

In conclusion, the supramolecular complex of FA‐MβCD with Ad‐HSA formed by host–guest interactions could significantly prolong the blood retention of FA‐MβCD and improve its antitumor effect and safety. Future studies should clarify the tumor tissue accumulation of FA‐MβCD after administration of this supramolecular complex and investigate its antitumor effect on various types of cancers. These findings provide useful basic data for the dynamic control of cancer cell‐selective anticancer agents through supramolecular complex formation using Ad‐HSA and CD.

## Conflict of interest

The authors declare no conflict of interest.

## Author contributions

TH and KM involved in conceptualization and methodology; YY, SM, NK, and RO involved in investigation; AS and KM involved in writing original draft preparation; AS, KM, and HA involved in writing review and editing; TH, KM, and HA involved in supervision. All authors have read and agreed to the published version of the manuscript. All authors have approved the final version of the manuscript.

## Data Availability

The data that support the findings of this study are available in the figures of the published article from the corresponding author Keiichi Motoyama upon reasonable request.
